# Apolipoprotein F concentration, activity, and the properties of LDL controlling ApoF activation in hyperlipidemic plasma

**DOI:** 10.1016/j.jlr.2021.100166

**Published:** 2022-01-08

**Authors:** Richard E. Morton, Daniel Mihna

**Affiliations:** Department of Cardiovascular and Metabolic Sciences, Lerner Research Institute, Cleveland Clinic Foundation, Cleveland, OH, USA

**Keywords:** apolipoproteins, plasma lipid transfer proteins, LDL metabolism, apolipoprotein F, cholesteryl ester transfer protein, lipid biochemistry, lipoproteins, hypercholesterolemia, hypertriglyceridemia, ApoB, apolipoprotein B, ApoF, apolipoprotein F, CE, cholesteryl ester, CETP, cholesteryl ester transfer protein, DPH, 1,6-diphenyl-1,3,5 hexatriene, FC, free cholesterol, FPLC, fast protein liquid chromatography, HyperTC, hypercholesterolemic, HyperTG, hypertriglyceridemic, LPC, lysophosphatidylcholine, PC, phosphatidylcholine, PL, phospholipid, TC, total cholesterol, TG, triglyceride, TMA-DPH, 1-(4-trimethylammoniumphenyl)-1,3,5-hexatriene

## Abstract

Apolipoprotein F (ApoF) modulates lipoprotein metabolism by selectively inhibiting cholesteryl ester transfer protein activity on LDL. This ApoF activity requires that it is bound to LDL. How hyperlipidemia alters total plasma ApoF and its binding to LDL are poorly understood. In this study, total plasma ApoF and LDL-bound ApoF were quantified by ELISA (n = 200). Plasma ApoF was increased 31% in hypercholesterolemic plasma but decreased 20% in hypertriglyceridemia. However, in donors with combined hypercholesterolemia and hypertriglyceridemia, the elevated triglyceride ameliorated the rise in ApoF caused by hypercholesterolemia alone. Compared with normolipidemic LDL, hypercholesterolemic LDL contained ∼2-fold more ApoF per LDL particle, whereas ApoF bound to LDL in hypertriglyceridemia plasma was <20% of control. To understand the basis for altered association of ApoF with hyperlipidemic LDL, the physiochemical properties of LDL were modified in vitro by cholesteryl ester transfer protein ± LCAT activities. The time-dependent change in LDL lipid composition, proteome, core and surface lipid packing, LDL surface charge, and LDL size caused by these factors were compared with the ApoF binding capacity of these LDLs. Only LDL particle size correlated with ApoF binding capacity. This positive association between LDL size and ApoF content was confirmed in hyperlipidemic plasmas. Similarly, when in vitro produced and enlarged LDLs with elevated ApoF binding capacity were incubated with LPL to reduce their size, ApoF binding was reduced by 90%. Thus, plasma ApoF levels and the activation status of this ApoF are differentially altered by hypercholesterolemia and hypertriglyceridemia. LDL size is a key determinate of ApoF binding and activation.

Cholesteryl ester transfer protein (CETP) promotes the net movement of cholesteryl ester (CE) and triglyceride (TG) between plasma lipoproteins (1, 2, 3, 4). Since most plasma CE is synthesized on HDL by LCAT, this creates a CE concentration gradient between lipoproteins. CETP functions to redistribute this CE to VLDL and LDL in exchange for TG in these lipoproteins.

Apolipoprotein F (ApoF) is a minor plasma protein that regulates CETP activity (5). ApoF inhibits CETP activity by preventing CETP from binding to the lipoprotein surface, an essential initial step in the lipid transfer process (6, 7). Most ApoF is contained in large HDL particles, but a portion also exists on LDL (8, 9). Only ApoF bound to LDL inhibits CETP activity, and there is an inverse linear relationship between the amount of ApoF on LDL and the capacity of that LDL to participate in CETP-mediated lipid transfer (9). This suggests that LDL-associated ApoF provides an important control point in the redistribution of CE from HDL into LDL versus VLDL and may control LDL-C levels. This concept is supported by recent studies in hamsters where ApoF knockdown increased LDL-C (10).

Importantly, the distribution of ApoF between the HDL-associated, inactive pool and the active LDL pool is under metabolic control (11). This redistribution is driven by compositional changes in LDL that influence its ability to bind ApoF rather than changes in the inactive pool that promote the release of ApoF. Properties of LDL that promote ApoF binding are not well understood but appear to correlate with the ratio of surface-to-core lipids (11). Notably, LDL from hypercholesterolemic (HyperTC) individuals with very high cholesterol levels contain more ApoF (9), suggesting that dyslipidemia may alter LDL composition and directly impact ApoF binding.

The effect of hyperlipidemia on plasma ApoF levels is not well understood. Only one study has investigated ApoF concentrations in humans with various hyperlipidemic conditions (12). Although some differences in ApoF levels between males and females and their response to hyperlipidemia were noted, ApoF levels between lipid phenotype groups were not different. The lack of an ApoF response to hypercholesterolemia in this study is unexpected since ApoF is increased 2-fold in HyperTC animals (13). The failure to detect changes in ApoF in these hyperlipidemic individuals may reflect the high percentage of subjects in this study with mildly elevated plasma lipids. Nevertheless, a negative correlation between ApoF and plasma TG levels and a positive correlation between HDL-C and ApoF were observed in male subjects.

In this study, we further examined how elevated plasma cholesterol and/or TG impact ApoF. ApoF was quantified by ELISA in normolipidemic, HyperTC, and hypertriglyceridemic (HyperTG) individuals plus in those with combined hypercholesterolemia and hypertriglyceridemia. Subjects with mild elevations in these lipids were excluded. We also determined how these hyperlipidemias impacted the activation status of ApoF through its binding to LDL and identified chemical and physical properties of LDL that influence ApoF binding. We found that hypercholesterolemia and hypertriglyceridemia uniquely alter both total ApoF levels and the portion of that ApoF that is LDL bound. Differences in ApoF binding are driven by changes in LDL size.

## Materials and methods

### Plasma samples

Plasma was obtained from two groups of donors. Stable patients undergoing elective cardiac evaluations at a tertiary referral center were invited to participate in an on-going study investigating factors contributing to the development of heart disease. Plasma was isolated from whole blood collected in EDTA tubes. Samples were maintained at 4°C immediately following phlebotomy, processed within 4 h of blood draw, and stored at −80°C until use. Subjects were fasting at the time of sample collection, and current medication, clinical information, and demographic information were collected. Anonymized (coded) archival aliquots of plasma were selected for study based on the inclusion/exclusion criteria described in Table 1. Alternatively, fresh plasma was obtained from recruited volunteers. Plasma was isolated from blood collected in the presence of citrate-dextrose anticoagulant. Plasma was stored at 4°C for short-term use. Informed consent was obtained predonation from all participants. The Institutional Review Board of Cleveland Clinic approved these study protocols. This work abides by the Declaration of Helsinki principles.

**Table 1:** Lipid group characteristics

Characteristic	All Subjects (*N* = 200)	Normolipidemic (*n* = 50)	HyperTC (*n* = 50)	HyperTG (*n* = 50)	HyperTC + TG (*n* = 50)	*P*
Age (year)	57.1 ± 13.1	58.5 ± 16.2	55.4 ± 12.0	58.1 ± 12.7	56.3 ± 11.1	ns
Male (%)	50.0	50.0	50.0	50.0	50.0	ns
Diabetes (%)	16.5	6.0	8.0	28.0	24.0	0.0039
Hypertension (%)	56.5	54.0	46.0	74.0	52.0	0.0297
Smoking (%)	33.3	35.4	34.0	48.0	16.0	0.0082
BMI (kg/m^2^)	28.8 (24.7–33.0)	26.8 (23.7–29.9)	26.6 (23.4–30.7)	31.4 (25.5–35.2)	30.7 (26.5–35.0)	<0.0001
TC (mg/dl)	217.5 (161.8–271.0)	151.0 (141.0–166.0)	261.5 (253.2–278.8)	168.5 (147.2–180.5)	289.5 (265.2–337.0)	<0.0001
TG (mg/dl)	174.5 (92.8–255.3)	85.5 (64.0–107.0)	102.0 (85.5–120.8)	242.0 (214.5–294.2)	260.0 (239.0–329.8)	<0.0001
LDL-C (mg/dl)	128.0 (83.8–183.8)	86.5 (70.0–98.8)	184.0 (169.0–199.8)	81.0 (62.5–90.5)	186.5 (168.0–212.0)	<0.0001
HDL-C (mg/dl)	43.0 (34.8–55.0)	47.5 (37.0–57.5)	61.0 (45.2–71.8)	32.5 (29.0–39.0)	45.0 (32.3–51.8)	<0.0001
Lipid-lowering medication (%)	42	0	46	60	62	<0.0001
Hypertension medication (%)	19	12	18	16	40	<0.0001
Diabetes medication (%)	10	4	6	14	18	<0.0001

ns, not significant.

Plasmas were selected based on the criteria indicated. Statistical analysis was performed as described in the Materials and methods section.

Normolipidemic: TC <200 mg/dl, LDL cholesterol <130 mg/dl, total TG <150 mg/dl. No lipid-lowering drugs (no statin, zetia, and niacin).

HyperTC: hypercholesterolemic (TC >240 mg/dl, LDL cholesterol >130 mg/dl, and TG <150 mg/dl).

HyperTG: hypertriglyceridemic (TC <200 mg/dl, LDL cholesterol <130 mg/dl, and TG >200 mg/dl).

HyperTC + TG: combined hypercholesterolemic and hypertriglyceridemic (TC >240 mg/dl, LDL cholesterol >130 mg/dl, and TG >200 mg/dl).

Age: mean ± SD; lipid values: median (interquartile range).

### ApoF ELISA

The immunoassay for plasma ApoF was performed essentially as previously described with two changes (12). A goat anti-ApoF primary antibody, instead of rabbit antibody, was used. Goat anti-ApoF was prepared commercially (Open Biosystems, Huntsville, AL) using the same recombinant ApoF-GST antigen as previously described for rabbit immunizations (12). In addition, antigen solubilization conditions were changed to improve the dose responsiveness of the assay for hyperlipidemic samples. Standards and samples were diluted with a PBS solution containing 1% BSA, 0.02% NaN_3_, 1% Tween-20 (catalog no.: K40000; surfactant no. 22), and 0.5% BRIJ 52 (catalog no.: K40000; surfactant no. 4) (QED Biosciences, Inc, San Diego, CA). Plasma samples were assayed at two dilutions (typically 1/80 and 1/160, but adjusted as needed). For the four lipid groups analyzed, the following were the ratios of ApoF concentrations determined at these two dilutions: normolipidemic: 0.999 ± 0.010 (mean ± SE); HyperTC: 0.974 ± 0.010; HyperTG: 0.981 ± 0.011; and combined HyperTC + HyperTG: 1.003 ± 0.013.

A plasma ApoF standard was created by pooling plasma from five normolipidemic donors, and its ApoF concentration was determined by MS as described in supplemental Data section using a heavy-labeled peptide internal standard approach. Aliquoted plasma standard was stored at −80°C. Standard plasma was serially diluted 1/40 to 1/320 and run as a standard curve on each 96-well plate. Each standard and sample dilution was assayed in triplicate. Data validating the specificity of the goat anti-ApoF antibody and ELISA are shown in supplemental Fig. S1. Both plasma forms of ApoF, reflecting different glycosylation status (12), are detected by the antibody.

### ApoF distribution in plasma

Plasma (250–500 μl) was fractionated on tandem Superose 6 columns, and 1 ml fractions were collected and assayed for their ApoF content by ELISA. As previously reported (9), two peaks of ApoF are observed—one coeluting with LDL and a second HDL-associated fraction with an apparent molecular mass of 470 kDa. This profile is stable for at least 2 months and for several freeze-thaw cycles when plasma is stored at −80°C. However, ApoF on LDL in plasma held at −80°C dissociates over time. At 26 weeks, approximately 20% of LDL-associated ApoF dissociates and is recovered in a novel ∼96 kDa peak. In long-term stored plasmas, very little ApoF remains on LDL. ApoF in the 470 kDa peak appears to be stable during storage. When analyzing the distribution of ApoF in frozen plasma samples, the amount of ApoF associated with LDL was taken as the sum of ApoF recovered in the LDL and 96 kDa peaks. Also, frozen plasma often contains particulate material due, in part, to VLDL aggregation. Particulates were removed before chromatographic analysis by 0.45 μm filtration (Millipore, Burlington, MA). Filtering did not remove detectable ApoF. Plasma cholesterol was reduced by <10%. However, this step did remove up to 50% of sample TG. During subsequent Superose 6 chromatography of filtered sample, the loss of cholesterol, TG, or ApoF was <10%.

### Modification of LDL

Lipoproteins and lipoprotein-deficient plasma (density >1.21 g/ml fraction) were isolated from fresh plasma by sequential ultracentrifugation (14) and extensively dialyzed against 0.9% NaCl, 0.02% NaN_3_, 0.02% EDTA, and pH 7.4. In some instances, the chemical and physical properties of LDL were modified in vitro by the coincubation of LDL and VLDL in the presence of CETP activity ± LCAT activity as previously described (11). A typical incubation contained ∼0.4 ml VLDL (1.45 mg TG), 0.6 ml LDL (2.3 mg cholesterol [TC]), plus 2.4 ml of freshly isolated lipoprotein-deficient plasma as a source of CETP and LCAT activities. Some samples received 1 mM paraoxon (Sigma) to inhibit LCAT activity. After incubation at 37°C for the indicated time, LDL was reisolated by sequential ultracentrifugation as the 1.019–1.063 g/ml density fraction (14) and dialyzed against 0.9% NaCl, 0.02% NaN_3_, 0.02% EDTA, and pH 7.4.

The capacity of modified LDL to bind ApoF was determined as previously described (11). For this, the 470 kDa complex containing inactive ApoF was purified from HDL_3_ by gel filtration chromatography (9). Subsequently, 470 kDa ApoF (50 μg protein) and 190 μl lipoprotein-deficient plasma were preincubated for 1 h at 37°C with 3.6 μg anti-CETP (TP2) (Ottawa Heart Institute, Ottawa, Ontario, Canada) and paraoxon (1 mM final) to inhibit endogenous CETP and LCAT activities. Then, modified LDL (120 μg protein) was added, and the incubation at 37°C continued for 6 h. Samples were fractionated by gel filtration fast protein liquid chromatography (FPLC) (9). ApoF in LDL and 470 kDa peaks were quantified by ELISA.

### Lipoprotein compositional analysis

Lipoprotein TC, free cholesterol (FC), and TG were quantified by enzyme-based kits from Thermo Fisher Scientific (Waltham, MA) (TC and TG) and Wako Diagnostics, Inc (Mountain View, CA) (FC). CE was calculated as TC minus FC times 1.69 to adjust for the fatty acid contained in this molecule. SM, phosphatidylcholine (PC), and lysophosphatidylcholine (LPC) were measured by kits (K600, K576, and K735, respectively) from BioVision, Inc (Milpitas, CA). Total phospholipid (PL) phosphorus was determined chemically (15). Protein was measured by a modification of the Lowry method (16). Apolipoprotein B (ApoB) was quantified by immunoassay (catalog no.: ab190806; Abcam, Waltham, MA) with human LDL as standard.

### LDL electrophoretic mobility

The relative electrophoretic mobility of LDL was determined on 1% agarose gels using the QuickGel Lipo Gel system (Helena Laboratories, Beaumont, TX). Samples were electrophoresed at 220 V for 25 min following the manufacturer's instructions. Gels were dried and stained with 0.1% Oil Red O in methanol.

### LDL size

LDL was chromatographed on tandem Superose 6 FPLC columns with on-line cholesterol detection as previously described (13). The absorbance (505 nm) readout was captured at 5 s intervals.

### Fluorescence polarization

The molecular packing of lipids in LDL was assessed with two fluorescent probes: 1-(4-trimethylammoniumphenyl)-1,3,5-hexatriene (TMA-DPH; Cayman Chemical, Ann Arbor, MI) and 1,6-diphenyl-1,3,5 hexatriene (DPH) (17). A 1 mM stock solution of TMA-DPH was prepared in ethanol and stored in the dark at −20°C. Working solutions were prepared in ethanol just before use. TMA-DPH was combined with LDL and incubated for 1 h at 37°C. The final solution contained <0.1% ethanol. Alternatively, a 1.72 mM stock solution of DPH (Sigma) was prepared in DMSO and stored at room temperature protected from light. The DPH stock solution was diluted in DMSO prior to use. DPH was combined with LDL and incubated for 1 h at 37°C. The final solution contained <0.1% DMSO. For both fluorophores, the mole ratio of added probe to LDL PL was 1:500.

Fluorescence was measured on a custom Photon Technology International (Birmingham, NJ) fluorimeter with fluorescence polarization accessory. A temperature-controlled cuvette chamber maintained samples at 37°C. Samples were excited at 360 nm, and excitation fluorescence was observed at 427 nm. Fluorescence anisotropy (r) was calculated by the equation: (I_vv_ − (G ∗ I_vh_))/(I_vv_ + (2GI_vh_)). G (grating factor) was calculated as I_hv_/I_hh_. I is the fluorescence intensity measured. Filter positions are indicated by subscripted letters. The first letter indicates the excitation filter position, and the second letter is the emission filter position (v= 0°; h= 90°).

### LC/MS

Samples were fractionated on 12% Criterion TGX precast gels (Bio-Rad Labs, Hercules, CA). Sample lanes on fixed and stained gels were divided into four smaller pieces. For protein digestion, gel pieces were washed with water, dehydrated in acetonitrile, then reduced with DTT, and alkylated with iodoacetamide. Proteins were digested in-gel using trypsin, by adding 5 μl of 10 ng trypsin/μl in 50 mM ammonium bicarbonate and incubating overnight at room temperature to achieve complete digestion. Peptides were extracted from the polyacrylamide with two aliquots of 50% acetonitrile with 5% formic acid. These extracts were combined and evaporated to <10 μl in a Speedvac and then resuspended in 1% acetic acid to make up a final volume of ∼30 μl for LC/MS analysis. The LC/MS system was a ThermoFisher LTQ-Orbitrap Elite hybrid mass spectrometer system. The HPLC column was a Dionex 15 cm × 75 μm id Acclaim Pepmap C18, 2 μm, 100 Å reversed phase capillary chromatography column. Five microliter volumes of the extract were injected, and the peptides eluted from the column by an acetonitrile/0.1% formic acid gradient (2–70%) at a flow rate of 0.3 μl/min were introduced into the source of the mass spectrometer online. The nano electrospray ion source is operated at 1.9 kV. The digest was analyzed using the data-dependent multitask capability of the instrument acquiring full scan mass spectra to determine peptide molecular weights and product ion spectra to determine amino acid sequence in successive instrument scans. This mode of analysis produces approximately 15,000 collisionally induced dissociation spectra of ions ranging in abundance over several orders of magnitude.

The data were analyzed using MaxQuant, version 2.0.1.0 with the search engine Andromeda, which is integrated into the MaxQuant software. Parameters used were default settings for the Orbitrap instrument. The database used to search the MS/MS spectra was the SwissProt human protein database containing 26,594 entries with an automatically generated decoy database (reversed sequences). The search was performed looking for fully tryptic peptides with a maximum of two missed cleavages. Oxidation of methionine and acetylation of protein N terminus were set as dynamic modifications, and carbamidomethylation of cysteine was set as static modifications. The precursor mass tolerance for these searches was set to 10 ppm, and the fragment ion mass tolerance was set to 0.6 Da. A false discovery rate was set to 1% with a minimum length of seven amino acids. Two unique or razor peptides were required for positive identification. Protein quantifications were performed with the label-free method available in the MaxQuant program (supplemental Table S1).

### Hydrolysis of LDL TG

To specifically hydrolyze TG, LDL (120 μg protein) was combined with 8 μl bovine milk lipoprotein lipase (catalog no.: L2254; Sigma, ≥2,000 units/mg) and 2.3% fatty acid-deficient BSA (Sigma) in 44 mM Tris-HCl, 150 mM NaCl, 0.02% NaN_3_, 0.02% EDTA, and pH 7.4. Lipase was not added to control LDL. After 1 h at 37°C, all samples received 1 mM paraoxon to quench lipase activity. The 470 kDa ApoF fraction (50 μg protein) was added, and incubation continued at 37°C for four additional hours to permit ApoF binding to LDL. Samples were fractionated by gel filtration FPLC, and the distribution of ApoF in LDL and 470 kDa fractions was determined by ELISA.

### Statistical analysis

Statistical analysis between two groups was performed by unpaired *t*-test or by Mann-Whitney nonparametric test if group variances were not equal (Instat 3; GraphPad Software, San Diego, CA). For multiple comparison between groups, statistical analysis was performed by ANOVA with Bonferroni's postcomparison test to determine adjusted *P* values (Prism; GraphPad Software, La Jolla, CA). In Table 1, *P* values were calculated by Kruskal-Wallis test for continuous data and Pearson's Chi-square test for categorical factors. The analyses were preformed using R 3.6.3 (Vienna, Austria; 2020). In all cases, *P* < 0.05 was considered statistically significant.

## Results

### ApoF levels in hyperlipidemic plasma

Subject population characteristics are shown in Table 1. Normal and hyperlipidemic plasmas were selected based on the criteria stated in the table. Subjects with mild hypercholesterolemia (200–240 mg/dl TC) or mild hypertriglyceridemia (150–200 mg/dl TG) were excluded from the study. Within a group, TC, TG, and LDL-C levels were not different between male and female subjects. As expected, female subjects had, on average, 12 mg/dl higher HDL-C than males in each group.

As assessed by ELISA, ApoF levels were not different between male and female subjects of a given lipid phenotype (Table 2). In HyperTC subjects, two distinct groups were observed, one where ApoF levels were elevated compared with control, and a second, smaller group where ApoF levels were very low (<10% of the group average). Subjects with very low ApoF are analyzed separately later. In the larger HyperTC group, ApoF levels were elevated 34%, which was largely because of a 42% increase in female donors ApoF compared with normolipidemic individuals. The increased ApoF in male HyperTC subjects did not reach statistical significance (*P* = 0.0990). In HyperTG female subjects, ApoF levels were reduced by 22% compared with normolipidemic females. However, among all HyperTG subjects, the reduced ApoF levels did not reach statistical significance (*P* = 0.0775). In subjects with both hypertriglyceridemia and hypercholesterolemia, ApoF levels were not different from normolipidemic controls. Notably, however, compared with individuals with hypercholesterolemia only, ApoF levels in both HyperTC + TG males and females were reduced by ∼25%. This shows that hypertriglyceridemia nullifies the ApoF-raising effect of hypercholesterolemia.

**Table 2:** ApoF levels in hyperlipidemic male and female subjects

Lipid Group	ApoF (μg/ml)		
	All	Male	Female
Normolipidemic	7.24 ± 0.35	7.20 ± 0.36	7.27 ± 0.57
HyperTC	9.67 ± 0.46^a^ (38)	8.97 ± 0.63 (19)	10.36 ± 0.65^a^ (19)
HyperTG	6.02 ± 0.30	6.37 ± 0.45	5.68 ± 0.39^b^
HyperTC + TG	7.32 ± 0.40^c^	6.78 ± 0.64^d^	7.91 ± 0.44^c^

Plasma ApoF concentrations were determined by immunoassay. Values are mean ± SE (n = 50 per lipid group or 25 per gender), unless indicated otherwise. See Table 1 for lipid group definitions. Statistical significance was assessed by ANOVA with Bonferroni's postcomparison test.

a *P* < 0.01 versus same normolipidemic group.

b *P* < 0.05 versus same normolipidemic group.

c *P* < 0.01 versus same HyperTC group.

d *P* < 0.05 versus same HyperTC group.

### HyperTC subjects with very low ApoF

**Fig. 1:**
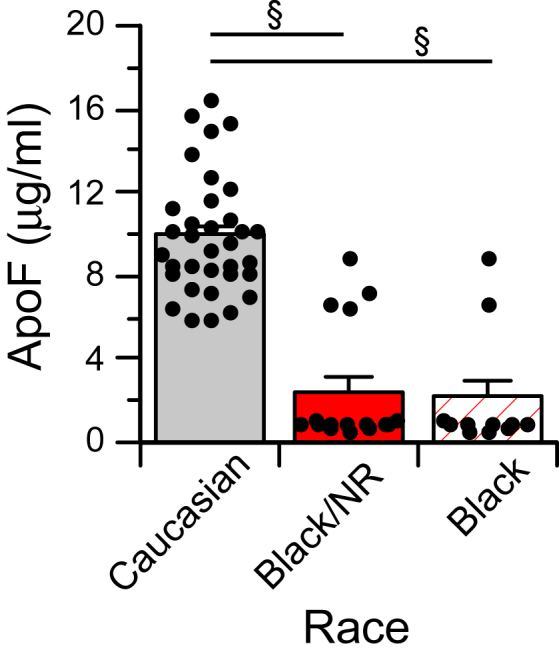
ApoF in HyperTC donors based on self-identified race. NR = no race identifier provided by donor. Values are the mean ± SEM of n = 34 (Caucasian), 16 (Black + NR), and 11 (Black) donors per group. §*P* < 0.001 compared with Caucasian subjects.

As noted previously, a subset (n = 12 of 50) of HyperTC subjects had very low ApoF. These low levels were not because of sample protein degradation as evidenced by SDS-PAGE banding patterns (supplemental Fig. S2A). Furthermore, the very low ELISA ApoF values for these samples were supported by Western blot (supplemental Fig. S2B). Surprisingly, all the subjects with very low ApoF levels self-identified their race as either Black or did not specify a race. Of 11 HyperTC subjects identifying as Black, nine had ApoF levels less than 10% of the group average (Fig. 1). Characteristics of this Black HyperTC donor population are shown in supplemental Table S2. While very similar to the HyperTC Caucasian donor population in many respects, plasma cholesterol was modestly lower and the percentage of individuals taking lipid-lowering or hypertension medications was much higher among Black donors. To identify the possible impact of these medication differences on ApoF levels, the effect of these medications was evaluated in the much larger Caucasian donor population. ApoF levels were not impacted by lipid-lowering hypertension or diabetic medications (supplemental Table S3). Thus, the very low ApoF levels in HyperTC Black donors do not appear to reflect their higher medication use. In addition to the HyperTC group, three other samples in the total study group (n = 200) had very low ApoF levels, one subject normolipidemic and two subjects with combined hypercholesterolemia and hypertriglyceridemia. All three self-identified as Black. Although, with the exception of the HyperTC group, very low ApoF levels only occurred in a small subset of Black donors, ApoF levels in Black individuals were much lower than those of Caucasians in all lipid groups except HyperTG subjects (Table 3).

**Table 3:** ApoF levels by donor race

Lipid Group	ApoF (μg/ml)		*P*
	Caucasian	Black	
Normolipidemic	7.47 ± 0.35 (44)	4.51 ± 0.78 (6)	0.0047
HyperTC	9.94 ± 0.49 (34)	2.14 ± 0.86 (11)	<0.0001
HyperTG	6.07 ± 0.32 (45)	5.60 ± 1.34 (3)	ns
HyperTC + TG	7.11 ± 0.41 (39)	4.05 ± 1.46 (5)	0.0202

ns, not significant.

ApoF levels in lipid groups comparing individuals self-identifying as Caucasian or Black race. Values are the mean ± SE of the indicated group size. See Table 1 for lipid group definitions. Statistical significance was assessed by *t*-test.

### ApoF correlation with plasma lipids

Given the considerable impact of donor race on ApoF levels, the effect of hyperlipidemia on plasma ApoF levels (Table 2) was re-evaluated in a donor subset containing only Caucasian subjects. Unlike the larger study groups (Table 2), BMI was not different between Caucasian lipid groups (not shown). ApoF was increased 31% in hypercholesterolemia but significantly decreased (20%) by hypertriglyceridemia (Table 4). For both HyperTC and HyperTG groups, alterations in ApoF levels were due to changes in female donors. Like that seen with the larger donor group (Table 2), the higher ApoF in HyperTC subjects was ameliorated by concomitant hypertriglyceridemia.

**Table 4:** ApoF levels in Caucasian male and female subjects

Lipid Group	ApoF (μg/ml)		
	All	Male	Female
Normolipidemic	7.60 ± 0.33 (44)	7.31 ± 0.36 (24)	7.83 ± 0.59 (20)
HyperTC	9.94 ± 0.49^a^ (34)	9.11 ± 0.71 (16)	10.67 ± 0.66^b^ (18)
HyperTG	6.07 ± 0.32^c^ (45)	6.33 ± 0.47 (24)	5.79 ± 0.43^c^ (21)
HyperTC + TG	7.11 ± 0.41^d^ (39)	6.48 ± 0.65^e^ (20)	7.78 ± 0.449^f^ (19)

Plasma ApoF concentrations were determined by immunoassay. Values are mean ± SE of the indicated group size. See Table 1 for lipid group definitions. Statistical significance was assessed by ANOVA with Bonferroni's post-test.

No differences between male and female subjects within a group by *t*-test.

a *P* < 0.001 versus same normolipidemic group.

b *P* < 0.01 versus same normolipidemic group.

c *P* < 0.05 versus same normolipidemic group.

d *P* < 0.001 versus same HyperTC group.

e *P* < 0.05 versus same HyperTC group.

f *P* < 0.01 versus same HyperTC group.

**Fig. 2:**
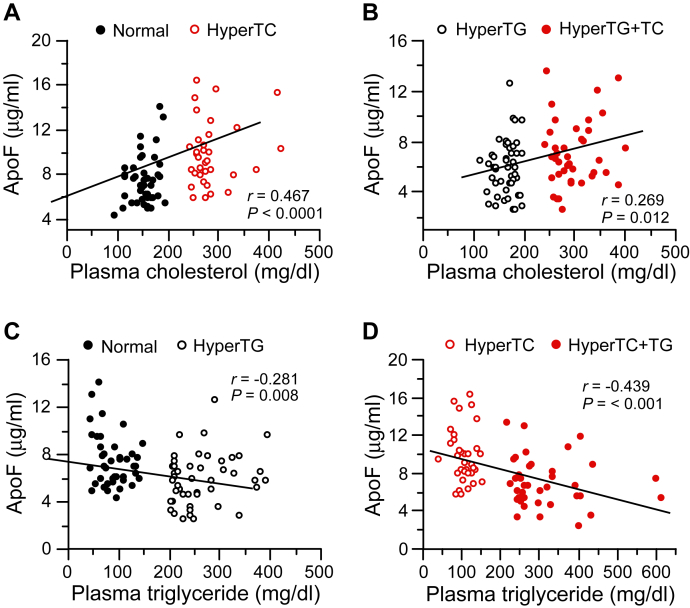
ApoF levels versus plasma cholesterol or TG concentration in Caucasian subjects. Plasma levels of ApoF are plotted versus plasma cholesterol levels in study subjects with normal (A) or elevated (B) levels of TG. Plasma levels of ApoF are plotted versus plasma TG levels in study subjects with normal (C) or elevated (D) levels of cholesterol. Lines of linear regression, correlation coefficient (*r*), and statistical probability (*P*) are shown.

Correlations between ApoF levels and plasma lipid levels were assessed in the Caucasian subgroup. When each lipid group was individually evaluated, in most cases, there was no statistically significant correlation between ApoF and plasma cholesterol or TG levels among all subjects in the group or for only male or female subjects. The sole exception was a positive relationship between plasma cholesterol and ApoF in normolipidemic female subjects (*r* = 0.545; *P* = 0.013). However, ApoF levels did correlate positively with plasma cholesterol when data for groups with the same TG (i.e., normolipidemic and HyperTC groups or HyperTG and HyperTC + TG groups) were analyzed (Fig. 2A, B). Conversely, ApoF levels correlated negatively with plasma TG when data for groups with the same cholesterol level (i.e., normolipidemic and HyperTG groups or HyperTC and HyperTC + TG groups) were analyzed. (Fig. 2C, D).

**Fig. 3:**
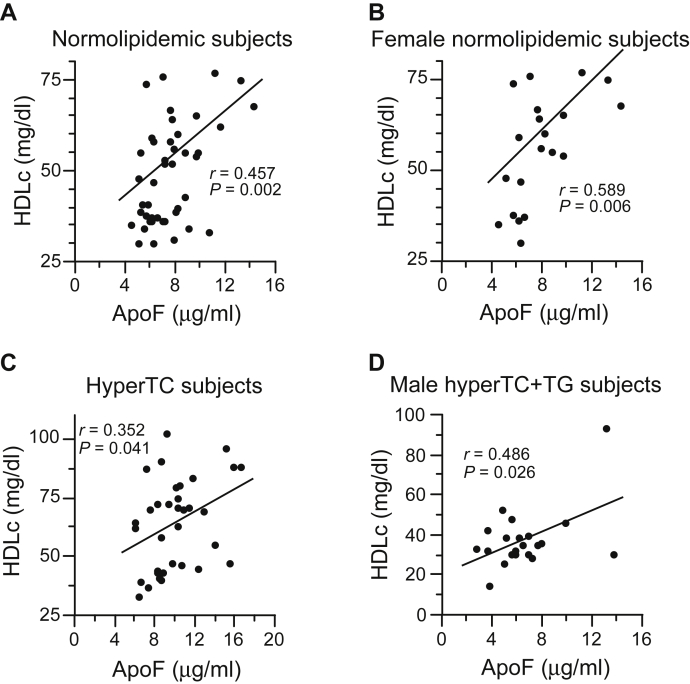
Correlation between HDL-C and ApoF levels in Caucasian subjects. A–D: Different study populations, as shown, were analyzed for the correlation between ApoF and HDL-C. Lines of linear regression, correlation coefficient (*r*), and statistical probability (*P*) are shown.

Our previous studies in hamsters observed that inhibition of ApoF expression caused HDL-C levels to fall, likely because of increased transfer of HDL-C CE to LDL-C in the absence of the inhibitory effects of ApoF on CETP transfer activity (10). To identify possible effects of ApoF levels on individual lipoproteins, we evaluated the effect of variable ApoF levels on plasma LDL-C and HDL-C in Caucasian subjects. Plasma LDL-C levels did not correlate with ApoF levels within a given group whether analyzed as a whole or genders separately. For HDL-C, correlation values are shown in supplemental Table S4. Plasma HDL-C correlated positively with ApoF levels in the normolipidemic group (Fig. 3A), which was due to an association in female donors (Fig. 3B), and in HyperTC subjects (Fig. 3C). An association between HDL-C with ApoF levels was not observed in HyperTG or HyperTC + TG groups but was observed in male HyperTC + TG subjects (Fig. 3D). The slopes of the four regression lines in Fig. 3 are not statistically different.

**Fig. 4:**
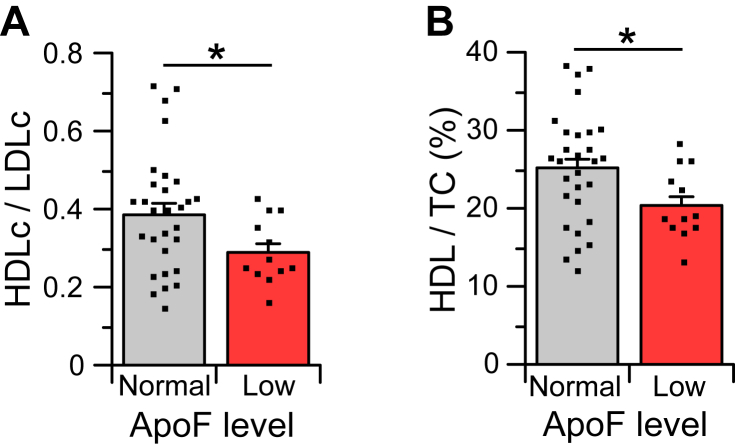
HDL-C levels in HyperTC individuals with typical versus very low ApoF. Plasma HDL-C/LDL-C (A) and percent of HDL-C/TC (B) values are compared in HyperTC individuals with very low ApoF levels (n = 12) and a subset of HyperTC individuals (n = 28) with typical ApoF levels and with plasma TC and TG levels similar to those in the ApoF-deficient group. Values are mean ± SEM. ∗*P* < 0.05 versus subjects with typical ApoF levels.

The presence of a subgroup of HyperTC donors with very low ApoF provides an additional opportunity to assess the association between plasma LDL-C or HDL-C and ApoF levels. For this analysis, a subgroup of Caucasian HyperTC subjects with the same plasma cholesterol range (245–298 mg/dl) as the subjects with very low ApoF was taken for comparison. TC and TG in these two groups were the same (264 vs. 259 mg/dl cholesterol, 100 vs. 101 mg/dl TG). LDL-C in ApoF-deficient subjects trended higher but did not quite reach statistical significance (*P* = 0.0519). However, plasma HDL-C levels in subjects with very low ApoF were significantly lower when expressed either as a ratio with LDL-C levels (Fig. 4A) or as a percentage of total plasma cholesterol (Fig. 4B). Overall, low ApoF levels are associated with a ∼13 mg/dl decrease in HDL-C.

### ApoF distribution in plasma

ApoF resides in two pools. One pool, contained in a 470 kDa subfraction of HDL, has no CETP inhibitory activity (9). The other pool of ApoF is associated with LDL, where it actively suppresses CETP activity. We previously reported that the ApoF content of LDL is increased in severe hypercholesterolemia (9). In recent studies with additional freshly obtained plasma samples, we confirmed that 11.9 ± 4.9% (n = 8) of plasma ApoF is bound to LDL in normolipidemic plasma, but 43.5 ± 15.8% (n = 5) is bound to LDL in HyperTC plasma. Although the concentration of LDL is higher in HyperTC plasma, this redistribution of ApoF in hypercholesterolemia causes a more than 2-fold increase in the amount of ApoF per LDL particle.

We have extended these studies by assessing the distribution of ApoF in the frozen archival plasmas studied previously. Because of the low volume of plasma available for each subject, plasma from five donors with similar cholesterol and TG content was pooled for analysis. Four to five such pools were created for each lipid group evaluated. The following was the average lipid content of these pools: normolipidemic (158 mg/dl TC, 91 mg/dl TG), HyperTC (272 mg/dl TC, 100 mg/dl TG), and HyperTG (162 mg/dl TC, 310 mg/dl TG). Pooled plasmas were fractionated by gel filtration FPLC, and the distribution of ApoF plasma lipoproteins was measured by ELISA. The lipid composition of FPLC-isolated LDL from these plasma pools is shown in supplemental Table S5. As seen with nonfrozen plasmas, the amount of ApoF associated with LDL in HyperTC plasma exceeds that seen with normolipidemic plasma (2.53 ± 0.35 μg ApoF/ml [n = 4] vs. 0.97 ± 0.33 μg ApoF/ml [n = 4], respectively [mean ± SE, *P* = 0.0176]). When expressed as ApoF per LDL particle (based on ApoB), the ApoF content of HyperTC LDL was 1.7-fold higher than normolipidemic LDL. Unexpectedly, in HyperTG plasma, LDL had very low ApoF content (0.15 ± 0.15 μg ApoF/ml [n = 5, *P* = 0.028]) compared with normolipidemic plasma. In fact, ApoF on LDL was undetectable in four of the five HyperTG plasma pools analyzed. Overall, these data show that more ApoF is active (LDL associated) in HyperTC subjects compared with control, whereas very little ApoF exists in the active form in HyperTG individuals.

### Analysis of LDL properties defining ApoF binding

We previously demonstrated that plasma ApoF moves from the inactive HDL-associated fraction to LDL during in vitro incubation of plasma at 37°C (11). In these incubations, CETP and LCAT activities in plasma altered the physicochemical properties of LDL. We concluded that the increased binding of ApoF by LDL correlates with LDL size, as approximated by the ratio of LDL core lipids to surface lipids. However, the specific size-dependent features of LDL that promote increased ApoF binding are not known. To gain further insight into the properties of LDL that control ApoF binding, we utilized a simplified protocol where LDL is premodified before being incubated with a source of ApoF (11). This permits detailed characterization of the chemical and physical properties of LDL that correlate with enhanced ApoF binding capacity.

These studies involve two steps. First, LDL is progressively modified by incubation with VLDL in the presence of CETP ± LCAT activity for varying times. CETP activity in these incubations promotes the exchange of TG in VLDL for CE in LDL causing the TG/CE ratio in LDL to increase. LCAT activity, on the other hand, produces CE, which blunts the rise in TG/CE, but it also consumes PL and FC. Following reisolation, these modified LDLs (supplemental Table S6) were incubated with the 470 kDa HDL subfraction enriched in ApoF. LDLs were reisolated by gel filtration FPLC, and the ApoF content of LDL was quantified by ELISA.

**Fig. 5:**
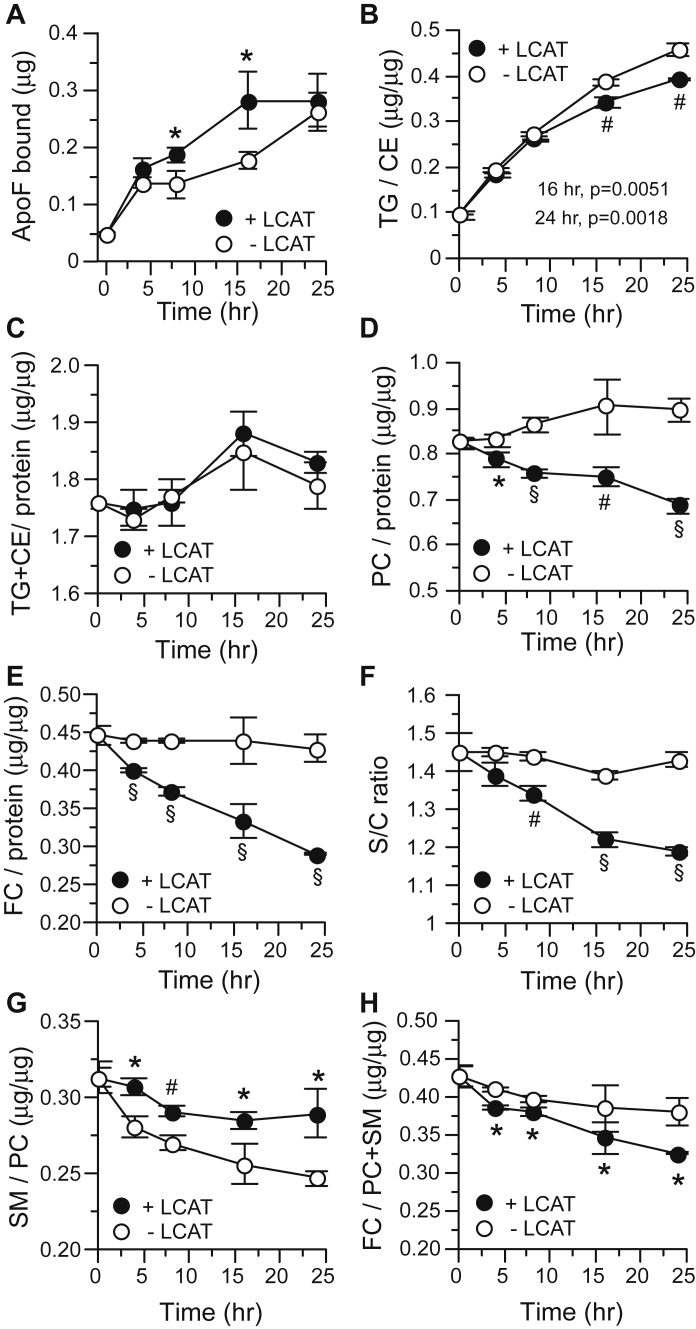
Changes in LDL composition over the time course of LDL modification by CETP ± LCAT activities. A: ApoF binding to modified LDL versus LDL modification time. B–H: Changes in LDL chemical composition during incubation. The S/C ratio shown in (F) is the sum of surface (S) components of LDL (protein + FC + PC + SM) divided by the sum of core (C) components (CE + TG). Values are the mean ± SD (n = 3) at each time point. ∗*P* < 0.05; ^#^*P* < 0.01; ^§^*P* < 0.001 versus no LCAT values at the same time.

The effect of incubation time on ApoF binding to LDL is shown in Fig. 5A. With long incubation times, the capacity of LDL modified with CETP activity alone or CETP + LCAT activities to bind ApoF is similar. However, at intermediate time points, the capacity of LDL modified under these two conditions to bind ApoF is different. There is no apparent correlation between incubation-induced changes in ApoF binding capacity and the levels of LDL components directly altered by CETP or LCAT activities, (i.e., TG/CE, TG + CE/protein, PC/protein, FC/protein) (Fig. 5B–E). Also, changes in the ratio of LDL surface (S = protein + FC + PC + SM) to core (C = CE + TG) components did not mirror ApoF binding (Fig. 5F). Among the lipids in the LDL surface, the ratio of SM/PC changed little over the incubation time course regardless of LCAT activity status and did not correlate with ApoF binding (Fig. 5G). The ratio of FC to PL (PC + SM) in the LDL surface decreased with time of incubation, with this decline being less when LCAT was inhibited (Fig. 5H). For all LDL modified ± LCAT, this change in FC/PC + SM correlated negatively with ApoF binding (*r* = −0.9068; *P* = 0.0007). Although LCAT activity creates LPC, LPC remained below the limit of detection in all modified LDL, likely because of its removal by albumin.

We quantified the proteome of modified LDL by MS to determine if the loss or acquisition of specific proteins is associated with enhanced ApoF binding capacity. Table 5 shows the proteins whose levels were significantly changed (≥2-fold, *P* < 0.05) in LDL following 24 h modification by CETP ± LCAT activities. The levels of 20 proteins were different in LDL modified by both CETP and LCAT activities compared with four proteins when modified by CETP alone. Since these LDLs have similar ApoF binding capacities (Fig. 5A), the 16 proteins whose levels statistically changed only when LCAT was active do not appear to contribute to ApoF binding capacity. However, the four proteins increased to similar levels in LDL modified by CETP regardless of LCAT activity status may represent proteins that either promote ApoF binding directly or proteins that function as placeholders on LDL that can be displaced from the lipoprotein surface once ApoF is added.

**Table 5:** Proteomic analysis of LDL

Category	Protein	Group 1/Control		Group 2/Control		Group 1/Group 2	
		LFQ Ratio	*P*	LFQ Ratio	*P*	LFQ Ratio	*P*
Changed in group 1 only	Alpha-1-acid glycoprotein 2	3.74	0.012	1.91	0.385	1.95	0.136
	Angiotensinogen	10.37	0.006	4.29	0.094	2.41	0.056
	Apolipoprotein A-IV	4.15	0.003	0.75	0.319	5.50	0.003
	Apolipoprotein C-II	3.86	0.005	5.16	0.059	0.74	0.477
	Apolipoprotein L1	7.65	0.001	1.18	0.445	6.46	0.002
	Ceruloplasmin	2.27	0.001	2.32	0.114	0.98	0.948
	CETP	110.4	0.048	1.75	0.620	62.9	0.049
	Complement C5	0.44	0.016	0.78	0.231	0.56	0.055
	Complement component C9	5.35	0.021	1.33	0.346	4.01	0.030
	Cystatin-C	0.38	0.018	0.44	0.108	0.87	0.822
	Fibrinogen alpha chain	0.48	0.027	0.81	0.474	0.59	0.217
	Neutrophil defensin 3	0.12	0.001	1.12	0.731	0.11	0.043
	LCAT	2.72	0.016	0.94	0.804	2.87	0.015
	Protein AMBP	0.42	0.020	0.50	0.101	0.83	0.676
	Serum amyloid A-4 protein	2.63	0.009	3.40	0.061	0.77	0.479
	Transthyretin	0.50	0.000	0.75	0.316	0.66	0.312
Changed in Groups 1 & 2	Apolipoprotein C-III	3.45	0.002	2.62	0.005	1.31	0.142
	Apolipoprotein C-IV	6.70	0.007	5.32	0.009	1.26	0.387
	Haptoglobin-related protein	4.59	0.002	3.92	0.006	1.17	0.390
	Serum amyloid A-1 protein	2.21	0.016	3.13	0.002	0.70	0.093

AMBP, alpha-1-microglobulin/bikunin precursor; LFQ, label-free quantification.

LDL was modified as described in the Materials and methods section. The protein content of control LDL and LDL modified for 24 h by both CETP and LCAT activities (group 1) or CETP activity alone (group 2) was determined by MS. Shown are the proteins whose levels changed at least 2-fold compared with control LDL with a *P* value of <0.05. Values are derived from analysis of three samples for each condition. Statistical significance was assessed by *t*-test.

**Fig. 6:**
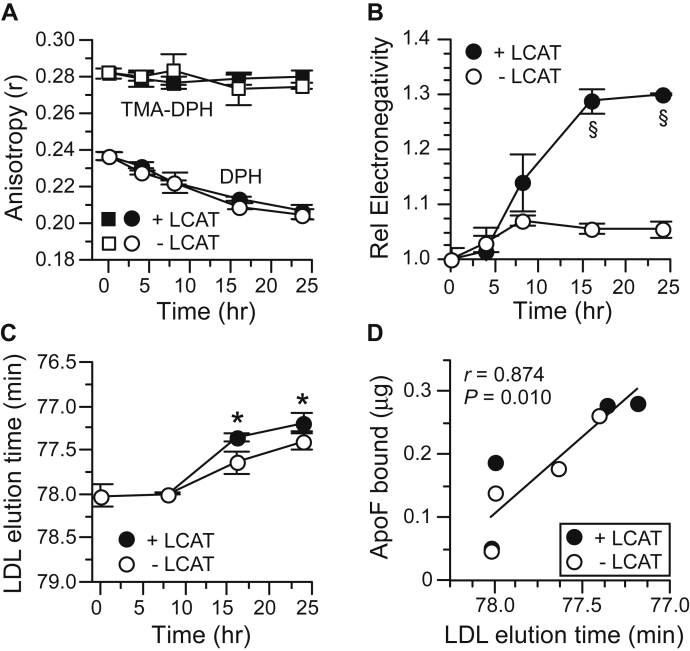
Changes in the physical properties of LDL over the time course of LDL modification by CETP ± LCAT activities. A: LDL lipid anisotropy. TMA-DPH reports lipid packing in the LDL PL surface. DPH reports the extent of lipid packing in the LDL core. B: Relative LDL electrophoretic mobility. C: LDL size assessed by gel filtration elution time. Note, the *y*-axis is reversed to directly reflect the increase in LDL size (shorter elution time) that occurs during the incubations. D: Correlation between ApoF binding and LDL size. Note, the *x*-axis is reversed to mirror (C). Values are the mean ± SD (n = 3) for each time point. ∗*P* < 0.05; ^§^*P* < 0.001 versus no LCAT values at the same time.

We also evaluated whether changes in the physical properties of LDL correlate with enhanced ApoF binding. The molecular packing of lipids in the core and surface of modified LDL was assessed by DPH and TMA-DPH anisotropy, respectively. TMA-DPH resides near in the surface of LDL and is sensitive to differences in the microviscosity of PL acyl chains and headgroups (18). DPH resides in the CE-TG rich core of LDL (19). There was no measurable change in surface lipid (TMA-DPH) anisotropy over time (Fig. 6A). This is surprising given that LCAT activity markedly changes the PC and FC in the LDL surface (Fig. 5). Core lipid (DPH) anisotropy decreased progressively with incubation time and equally in LDL modified ± LCAT activity. This likely reflects the similar CETP-driven change in LDL TG/CE ratio under these two conditions. Changes in the molecular packing of LDL lipids do not correlate with altered ApoF binding capacity.

It has been reported that ApoF is more abundant on a subpopulation of LDL with higher electronegativity (20). Modification of LDL by both CETP and LCAT activities produced LDL of higher electrophoretic mobility, but this change in LDL surface charge was largely prevented when LCAT activity was blocked (Fig. 6B). LDL surface charge does not appear to contribute to ApoF binding since LDL extensively modified ± LCAT activity have greatly different surface charges yet have similar ApoF binding capacity. However, changes in LDL particle size, as assessed by gel filtration chromatography (Fig. 6C, D), do correlate well with ApoF binding capacity (Fig. 5A). Larger LDLs (shorter elution time) have greater ApoF binding capacity. Limitations of this gel filtration method may preclude seeing LDL size differences earlier in the incubation time course (i.e., 8 h) where ApoF binding capacities are different when LCAT is active versus inactive (Fig. 5A).

### ApoF binding versus LDL size in hyperlipidemia

**Fig. 7:**
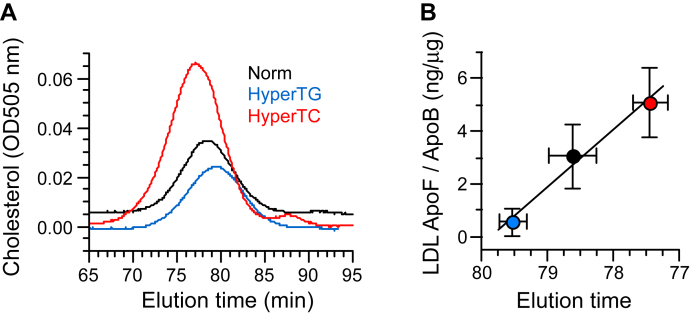
Correlation between LDL size and ApoF binding in hyperlipidemic plasmas. The size of LDL in hyperlipidemic and normolipidemic plasmas was determined by gel filtration FPLC. A: Typical LDL elution profiles for LDL. B: Correlation between the size of LDL from normolipidemic, HyperTC, and HyperTG subjects, as determined by FPLC, and the amount of ApoF per LDL particle expressed as the ratio of ApoF/ApoB. Shown are the mean ± SEM of n = 4–5 plasma pools for each lipid group. Note that the *x*-axis is reversed so that LDL size increases from left to right.

To examine further the relationship between LDL size and ApoF binding, we investigated whether the altered binding of ApoF to LDL observed previously in pooled hyperlipidemic plasmas might also reflect LDL particle size in these samples. Representative gel filtration profiles are shown in Fig. 7A. Compared with the elution time for normolipidemic LDL (78.61 ± 0.35 min [n = 3]), HyperTC LDL eluted at 77.44 ± 0.26 min (n = 3, *P* = 0.0097) versus normolipidemic LDL, indicating they are larger. Conversely, HyperTG LDLs are smaller, eluting at 79.52 ± 0.20 min (n = 3, *P* = 0.0174) versus normolipidemic LDL. The amount of ApoF bound per LDL particle in these plasmas correlated well with LDL size (Fig. 7B). These data provide further support for a direct relationship between LDL particle size and ApoF binding.

### Reverse modification of LDL

During the in vitro modification of LDL described previously, LDL became enlarged and had enhanced capacity to bind ApoF. To further examine the role of LDL particle size in ApoF binding, we investigated the effect of reducing the size of this LDL on ApoF binding. LDL, which has been modified by 24 h incubation with VLDL and only CETP activity (Fig. 5 and supplemental Table S6) to enrich its TG content, was incubated with lipoprotein lipase in the presence of fatty acid-free BSA to facilitate the hydrolysis of TG. After inhibiting lipase activity, the 470 kDa HDL subfraction enriched in ApoF was added. LDL was isolated from the mixture by gel filtration FPLC, and its ApoF content was determined by ELISA. Under these conditions, 36% of LDL TG was hydrolyzed, whereas the levels of other lipid components were unchanged (Table 6). This resulted in smaller LDLs that are similar in size to LDLs from HyperTG plasma. These smaller LDLs had markedly reduced capacity to bind ApoF. Notably, the ratio of FC to PL, which correlated negatively with ApoF binding in the aforementioned in vitro studies (Fig. 5H), was not changed by lipase treatment.

**Table 6:** Effect of TG hydrolysis on LDL size and ApoF binding capacity

LPL Treatment	*Weight Ratio*					LDL Elution (min)	ApoF Binding (%)
	TG/ApoB	CE/ApoB	PL/ApoB	TG/CE	FC/PL		
−	0.81 ± 0.04	1.83 ± 0.12	1.12 ± 0.06	0.44 ± 0.01	0.28 ± 0.03	78.18 ± 0.06	71.6 ± 11.8
+	0.52 ± 0.01^a^	2.00 ± 0.05	1.01 ± 0.04	0.26 ± 0.01^a^	0.28 ± 0.01	79.22 ± 0.06^a^	8.3 ± 7.2^a^

LDL, which had been enriched in TG by incubation with VLDL and CETP activity as described in the Materials and methods section, was incubated ± LPL to hydrolyze TG. After inhibiting LPL, LDLs were incubated with a source of ApoF and then reisolated by gel filtration FPLC. The chemical composition, particle size, and ApoF binding capacity of LDLs were determined as described in the Materials and methods section. Values are mean ± SD, n = 3. Statistical significance was assessed by *t*-test.

a *P* < 0.01 versus no LPL control.

## Discussion

Little is known about how ApoF levels are influenced by hyperlipidemia. ApoF levels in HyperTG subjects have been reported to be unchanged (12) or modestly reduced (21). Diet-induced hypercholesterolemia in rabbits and hamsters increases plasma ApoF 2-fold (13). However, in HyperTC humans, ApoF levels were not found to be elevated (12). However, this may be because a significant portion of these HyperTC subjects has mild hypercholesterolemia. To optimize conditions for identifying possible differences in ApoF levels among various dyslipidemic subjects, here we quantified plasma ApoF in three hyperlipidemic groups where individuals with clinically defined borderline elevations in TG or TC have been excluded. In contrast to previous studies, we observed that ApoF is increased markedly in Caucasian HyperTC subjects, which was largely because of the increase occurring in female subjects. ApoF was reduced in Caucasian HyperTG subjects, again because of the response of female subjects. In both male and female subjects with combined hypercholesterolemia and hypertriglyceridemia, ApoF levels were not different from normolipidemic controls. However, ApoF was decreased compared with subjects with hypercholesterolemia alone. This shows that hypertriglyceridemia ameliorates the rise in ApoF caused by hypercholesterolemia, consistent with that previously demonstrated by an independent approach (22). Overall, ApoF levels correlated positively with plasma cholesterol levels and negatively with plasma TG levels, supporting associations previously reported (12, 21).

Surprisingly, ApoF in Black donors was lower than in Caucasian donors in most all donor lipid groups. This was most acutely evident in HyperTC subjects where ApoF levels in most Black participants were less than 10% of the Caucasian participants. To our knowledge, this is the first report of race differences in ApoF levels. A study specifically designed to investigate this more thoroughly is needed.

ApoF preferentially inhibits CETP activity on LDL (23). This likely alters LDL synthesis and controls the flow of CE from HDL to LDL (24). In hamsters made deficient in ApoF, LDL-C levels increase and HDL-C levels fall (10). Although we found no association between LDL-C levels and ApoF in the lipid phenotypes studied, perhaps because of confounding influences of lipid-lowering drugs, we did observe significant positive correlations between ApoF levels and plasma HDL-C in several study groups. Conversely, in a subset of HyperTC subjects with very low ApoF levels, plasma HDL-Cs were reduced. Overall, these findings strongly suggest that ApoF plays a direct role in HDL-C levels.

The preferential inhibitory effect of ApoF on CETP activity with LDL (2, 23, 25) is because the active form of ApoF resides primarily on LDL (9). The extent to which LDL participates in CETP-mediated lipid transfer reactions is inversely related to its ApoF content (9). The association of ApoF with LDL is driven by the capacity of LDL to bind ApoF, not by the release of ApoF from the inactive pool (11). We report here that HyperTC LDLs are enriched in ApoF, expressed either as the fraction of plasma ApoF on LDL or as the amount of ApoF per LDL particle. HyperTG LDLs are ApoF deficient. This shows that the activation status of ApoF varies in hyperlipidemic subjects and may be increased or decreased independent of total plasma ApoF. Previous in vitro studies suggest that ApoF binding correlates positively with the ratio of core lipids to surface lipids in LDL (11), which generally equates with LDL size. However, multiple lipoprotein features such as surface charge density, the PL to protein ratio, the SM to PC ratio, and TG/CE are known to vary with ApoB-lipoprotein size (18, 26, 27, 28). Somewhat surprisingly, in our in vitro LDL modification system, none of these properties individually correlated with ApoF binding. ApoF binding did correlate with the ratio of FC/PL in this study, but this was not observed with LDL from hyperlipidemic subjects or with LDL modified by lipoprotein lipase in vitro, suggesting that this correlation is unique to this modification system. It is notable that the ratio of FC/PL in these in vitro modified LDL is highly correlated with LDL size (*r* = 0.8357; *P* = 0.0192).

Overall, the ability of LDL to bind ApoF is best explained by the physical size of LDL. This correlation was observed with in vitro modified LDL, LDL modified by lipoprotein lipase, and LDL isolated from hyperlipidemic plasmas. Lipoprotein size directly impacts the physical packing of lipids in the lipoprotein surface. This may occur because of constraints caused by surface curvature and/or subsequent changes in the composition of the lipid surface. The physical state of lipids in the lipoprotein surface is known to play an important role in the binding of multiple apolipoproteins (17). Interestingly, even though LDLs modified in vitro by coincubation with VLDL in the presence of CETP ± LCAT activities have large changes in surface lipids, the molecular packing of these lipids was not observed to be altered. This likely occurs because, as the lipid surface of LDL is modified during these incubations, additional proteins are recruited from VLDL to the LDL surface to fill molecular “gaps.” Proteomic analysis identified four proteins (ApoC-III, ApoC-IV, serum amyloid A-1, and haptoglobin-related protein) that are similarly enriched on LDL when it is extensively modified by either CETP alone or by CETP + LCAT activities. LDLs modified under these two conditions have similar ApoF binding capacities. It is possible that ApoF competes effectively with one or more of these acquired proteins for residence on the LDL surface once ApoF is added to the incubation. It is notable that LDL is enriched in additional proteins when extensively modified by both CETP and LCAT activities. However, ApoF binding to LDL containing or lacking these extra proteins is similar. Therefore, not all proteins acquired by LDL alter its ApoF binding potential. No protein was observed to be decreased in both LDL modified by CETP alone and in LDL modified by CETP + LCAT activities. So, it is unlikely that a resident LDL protein blocks ApoF binding.

The surface lipids on LDL are much more ordered (low fluidity) than on its larger precursor, VLDL (18). Because ApoF is not commonly associated with VLDL, these findings suggest that as the physical packing of surface lipids increases during VLDL catabolism, remnant particles become suitable binding sites for ApoF. ApoF binding capacity decreases as these remnants reach typical LDL size and is lost completely if LDL become atypically small, as happens in hypertriglyceridemia.

The increased ApoF levels in HyperTC subjects observed here is consistent with the elevated ApoF levels observed in HyperTC rabbits and hamsters (13). However, ApoF gene expression, which occurs solely in the liver (13), is decreased in these animals. Also, treatment of human liver cells with cholesterol decreases both ApoF gene expression and ApoF secretion (13). One possible explanation for the disparity between ApoF synthesis and ApoF plasma levels in hypercholesterolemia is altered turnover of plasma ApoF. We speculate that the increased association of ApoF with LDL in hypercholesterolemia may retard ApoF clearance. This suggests that most ApoF exits the plasma compartment via the inactive 470 kDa fraction. A similar clearance mechanism has been reported for LCAT, which also exists in plasma in active and inactive forms. The removal of LCAT from plasma occurs primarily via the inactive complex (29).

In summary, we previously demonstrated that ApoF suppresses CETP activity and that this inhibition of CETP selectively occurs with lipid transfers involving LDL (24). In ApoF-deficient hamsters, plasma LDL-C levels increase and HDL-C levels decrease (10). We report here that ApoF protein levels are increased in HyperTC humans but decreased in hypertriglyceridemia. Hypertriglyceridemia also normalized ApoF levels in subject with elevated TC. ApoF levels were positively correlated with plasma HDL-C, consistent with the role of ApoF in regulating the removal of CE from HDL. We report for the first time that ApoF levels are lower in Black compared with Caucasian donors. Factors accounting for this difference remain undefined. Interestingly, the fraction of plasma ApoF that is active (i.e., bound to LDL) is high in hypercholesterolemia but very low in hypertriglyceridemia. LDL size is the major determinate of this difference, which impacts the molecular packing of surface phospholipids. Therefore, ApoF binding may be regulated by mechanisms similar to those controlling binding of apolipoproteins A-I, A-II, C-II, C-III, and A-IV (30, 31, 32). Changes in LDL size often correlate with incidence of disease (33, 34). Factors controlling LDL size may directly impact LDL-C and HDL-C levels by altering the binding of ApoF to LDL, which may directly control the flow of HDL CE to LDL by CETP.

## Data availability

The MS proteomics data have been deposited to the ProteomeXchange Consortium via the PRIDE partner repository with the dataset identifier PXD029713. All other data are contained within the article and supplemental data file.

## Supplemental data

This article contains supplemental data (8, 9, 12).

## Conflict of interest

The authors declare that they have no conflicts of interest with the contents of this article.
