# Supplementary material for: Apolipoprotein F concentration, activity, and the properties of LDL controlling ApoF activation in hyperlipidemic plasma
Source: J Lipid Res. 2022 Jan 8;63(2):100166. doi: 10.1016/j.jlr.2021.100166 (PMC8953654; doi:10.1016/j.jlr.2021.100166)
Supplement: Supplemental Tables S2–S6 and Figures S1 and S2 [file mmc1.docx]

**Supplemental Material**

Apolipoprotein F concentration, activity, and the properties of LDL controlling ApoF activation in hyperlipidemic plasma

Richard E. Morton and Daniel Mihna

Supplemental Method

**ApoF ELISA standard calibration -** Plasma from 5 normolipidemic individuals were pooled to create an ELISA standard. The ApoF content of this pool was determined by mass spectrometry using an internal standard approach based on a peptide that is generated by tryptic digestion of ApoF. The selected ApoF peptide, SGVQQLIQYYQDQK (peptide 1) and its heavy isotope analog, SGVQQLIQYYQDQ{Lys(13C6,15N2)} (peptide 2), were synthesized by GenScript, Piscataway, NJ). To determine the concentration of peptide solutions, peptides were subjected to vapor phase hydrolysis following by analysis on the AccQ-tag amino acid analysis system by Waters Corporation (Milford, MA)).

To quantify the ApoF content of the plasma standard, five μl of pooled plasma were combined with 45 μl 8M urea Tris-HCl pH 8 and protease inhibitor was added. Protein concentrations were estimated using the BCA method (Thermo Fisher Scientific, Waltham, MA). Ten μg of protein were taken for in-solution trypsin digestion with 15 μl of 10 fmole/μl synthetic peptide 2 spiked in. Samples were reduced with DTT and alkylated with iodoacetamide prior to the addition of 0.125 μg of trypsin and the proteins were digested overnight at room temperature to produce sample-derived peptide 1. The digest was stopped by the addition of 1 μl TFA, and the digested samples were desalted using C18 Ziptips and dried on a SpeedVac. The dried sample was reconstituted with 30 μl formic acid for LC-MS analysis.

The LC-MS system was a ThermoFisher Fusion Lumos mass spectrometer system. The HPLC column was a Dionex 25 cm x 75 μm id Acclaim Pepmap C18, 2μm, 100 Å reversed phase capillary chromatography column. Five μl volumes of the extract were injected and the peptides eluted from the column by an acetonitrile/0.1% formic acid gradient at a flow rate of 0.3 μl/min were introduced into the source of the mass spectrometer on-line. The nano electrospray ion source is operated at 2.3 kV. The digest was analyzed using a targeted acquisition method. The target peptides included +3 charged peptides and +2 charged peptides of both sample-derived peptide 1 and the internal standard peptide 2.

The data were analyzed manually by plotting the extracted ion chromatograms for the 4 targeted ions in each sample, and absolute quantities were calculated using calibration curves of synthetic peptide 1 (0 – 12.5 fmole/μl) containing 5 fmole/μl peptide 2. The ApoF fmoles/μl concentration of the plasma standard was recalculated as μg ApoF /ml plasma using an ApoF molecular weight of 17,425 (1).

Supplemental Data

**Table S1 -** Mass spectrometry quantification of LDL proteins. See the attached spreadsheet showing accession number, the number unique peptides detected, the percentage of sequence coverage, and relative abundance values for each identified protein.

**Table S2 –** Characteristics of Black and Caucasian hypercholesterolemic subjects. Unless indicated otherwise, values are mean ± SEM of the indicated group size. Statistical analysis was performed by Mann-Whitney test for continuous data and Fisher’s exact test for categorical factors.

|  |  |  |  |  |
| --- | --- | --- | --- | --- |
| Characteristic | | Black (11) | Caucasian (34) | *P* |
| Age | | 51.4 ± 2.8 | 55.7 ± 2.1 | ns |
| Male (%) | | 63.6 | 50.0 | ns |
| BMI (kg/m^2^) | | 29.2 ± 1.6 | 26.2 ± 0.9 | ns |
| TC (mg/dl) | | 253 ± 2 | 281 ± 8 | < 0.05 |
| TG (mg/dl) | | 97 ± 8 | 103 ± 4 | ns |
| LDLc (mg/dl) | | 179 ± 4 | 196 ± 9 | ns |
| HDLc (mg/dl) | | 54 ± 4 | 64 ± 19 | ns |
| Lipid-lowering med. (%) | | 82 | 32 | < 0.05 |
| Hypertension med. (%) | | 55 | 27 | < 0.05 |
| Diabetes med. (%) | | 9 | 18 | ns |

**Table S3 –** Effect of medications taken by donors on ApoF levels. ApoF levels (μg/ml) in Caucasian subjects were assayed by ELISA. Values are the mean ± SEM of the indicated group size. *P* values were determined by t-test between values ± the indicated medication.

|  | Lipid-lowering Medications | | |  | Diabetic Medications | | |  | Hypertensive Medications | | |
| --- | --- | --- | --- | --- | --- | --- | --- | --- | --- | --- | --- |
| Group | – | + | *P* |  | – | + | *P* |  | – | + | *P* |
| HyperTC | 9.86 ± 0.63 (23) | 10.11 ± 0.66 (11) | ns |  | 10.10 ± 0.54 (32) | 8.91 ± 0.24 (2) | ns |  | 9.82 ± 0.68 (25) | 9.18 ± 1.17 (9) | ns |
|  |  |  |  |  |  |  |  |  |  |  |  |
| HyperTG | 5.73 ± 0.42 (20) | 6.35 ± 0.47 (25) | ns |  | 6.11 ± 0.39 (32) | 6.17 ± 0.59 (11) | ns |  | 5.95 ± 0.51 (13) | 6.13 ± 0.40 (32) | ns |
|  |  |  |  |  |  |  |  |  |  |  |  |
| HyperTC+TG | 6.49 ± 0.63 (16) | 7.54 ± 0.54 (23) | ns |  | 6.83 ± 0.45 (32) | 8.40 ± 0.94 (7) | ns |  | 6.56 ± 0.64 (19) | 7.64 ± 0.56 (20) | ns |

**Table S4 -** Correlation (r) between plasma HDLc and ApoF levels in Caucasian subjects from the indicated lipid group. *P* values were determined by linear regression.

|  |  | Subjects | | |
| --- | --- | --- | --- | --- |
| Group | value | All | Male | Female |
|  |  |  |  |  |
| Normo | r | **0.457** | 0.135 | **0.589** |
|  | *P* | **0.002** | 0.521 | **0.006** |
|  |  |  |  |  |
| HyperTC | r | **0.352** | 0.234 | 0.338 |
|  | *P* | **0.044** | 0.335 | 0.157 |
|  |  |  |  |  |
| HyperTG | r | -0.085 | -0.019 | 0.289 |
|  | *P* | 0.558 | 0.929 | 0.152 |
|  |  |  |  |  |
| HyperTC+TG | r | 0.245 | **0.485** | -0.211 |
|  | *P* | 0.132 | **0.026** | 0.386 |

**Table S5 -** Lipid composition of human plasma LDL. Pooled normolipidemic and hyperlipidemic plasmas were fractionated by gel filtration. LDL isolated by this method was chemically characterized. Abbreviations – Norm, normolipidemic; HyperTC, hypercholesterolemic; HyperTG, hypertriglyceridemic; FC, free cholesterol; CE, cholesteryl ester; TG, triglyceride; PL, phospholipid; ApoB, apolipoprotein B. Values are the mean ± SD of 3 plasma pools for each lipid group. ^a^ *P* < 0.05 versus normolipidemic plasma, ^b^ *P* < 0.01 versus normolipidemic plasma.

| Lipid group | FC | CE | TG | PL |  |  |
| --- | --- | --- | --- | --- | --- | --- |
|  | μg/μg ApoB | | | | TG/CE | FC/PL |
| Norm | 0.47 ± 0.02 | 2.34 ± 0.26 | 0.30 ± 0.03 | 1.52 ± 0.17 | 0.13 ± 0.02 | 0.32 ± 0.04 |
| HyperTC | 0.48 ± 0.01 | 2.23 ± 0.01 | 0.22 ± 0.02 | 1.40 ± 0.07 | 0.09 ± 0.01 | 0.35 ± 0.02 |
| HyperTG | 0.36 ± 0.02^b^ | 2.20 ± 0.04 | 0.60 ± 0.16^a^ | 1.31 ± 0.06 | 0.28 ± 0.08^a^ | 0.28 ± 0.02 |

**Table S6 -** Lipid composition of modified LDL. LDL was incubated with VLDL and CETP ± LCAT for the indicated times as described in the Methods, and then reisolated by ultracentrifugation. FC- free cholesterol, CE cholesteryl ester, TG- triglyceride, PC- phosphatidylcholine, SM- sphingomyelin. Lyosphosphatidylcholine was not detectable in the samples. The lysophosphatidylcholine assay sensitivity was < 0.015 μg/μg LDL protein, or, < 1.5% of total LDL phospholipid. Values are mean ± SD, n = 3. ^a^ *P* < 0.05 versus t = 0 control, ^b^ *P* < 0.01 versus t = 0 control, ^c^ *P* < 0.05 versus no LCAT at same time point, ^d^ *P* < 0.01 versus no LCAT at same time point.

| Time (hr) | LCAT active | FC | CE | TG | PC | SM |
| --- | --- | --- | --- | --- | --- | --- |
|  |  | μg/μg protein | | | | |
| 0 | no | 0.466 ± 0.013 | 1.604 ± 0.063 | 0.157 ± 0.008 | 0.828 ± 0.011 | 0.259 ± 0.005 |
| 4 | no | 0.439 ± 0.003*^a^* | 1.451 ± 0.017*^a^* | 0.281 ± 0.004*^b^* | 0.834 ± 0.014 | 0.233 ±  0.004*^b^* |
| 8 | no | 0.439 ± 0.004*^a^* | 1.389 ± 0.004*^b^* | 0.379 ± 0.005*^b^* | 0.868 ± 0.017*^a^* | 0.234 ± 0.002*^b^* |
| 16 | no | 0.439 ± 0.031 | 1.334 ± 0.045*^b^* | 0.516 ± 0.026*^b^* | 0.907 ± 0.059 | 0.232 ± 0.008*^b^* |
| 24 | no | 0.429 ± 0.017*^a^* | 1.225 ± 0.028*^b^* | 0.561 ± 0.018*^b^* | 0.900 ± 0.026*^a^* | 0.222 ± 0.006*^b^* |
|  |  |  |  |  |  |  |
| 4 | yes | 0.400 ± 0.003*^bd^* | 1.480 ± 0.025*^a^* | 0.272 ± 0.009*^b^* | 0.790 ± 0.017*^ac^* | 0.242 ± 0.005*^a^* |
| 8 | yes | 0.373 ± 0.005*^bd^* | 1.391 ± 0.005*^b^* | 0.366 ±  0.008*^b^* | 0.759 ± 0.009*^bd^* | 0.221 ± 0.005*^bc^* |
| 16 | yes | 0.334 ± 0.022*^bd^* | 1.399 ± 0.029*^b^* | 0.483 ± 0.017*^b^* | 0.752 ± 0.020*^bc^* | 0.214 ± 0.008*^b^* |
| 24 | yes | 0.290 ± 0.002*^bd^* | 1.315 ± 0.015*^bd^* | 0.520 ± 0.010*^bc^* | 0.689 ± 0.017*^bd^* | 0.199 ± 0.007*^bc^* |


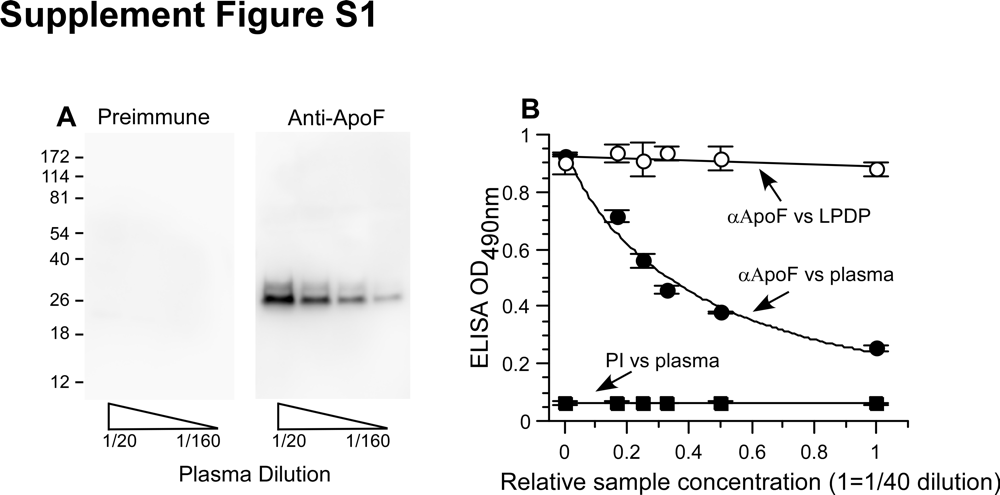


**Supplemental Figure S1** - Specificity of the immunoassay for ApoF. Panel A: Comparison of the immunoreactivity of goat preimmune and anti-ApoF sera. The indicated dilutions of human plasma were separated on 4-20% SDS-PAGE gels. Immunoblots were performed with goat preimmune or anti-ApoF sera diluted 1/1250 as the primary antibody. Panel B: ApoF ELISA assays were performed with the indicated dilutions (1/40 to 1/240) of human plasma or lipoprotein-deficient plasma (LPDP, density > 1.21 g/ml fraction). LPDP, which has no detectable ApoF (2, 3) but contains most other proteins in plasma, serves as a negative control. Goat preimmune (PI) or anti-ApoF (αApoF) sera were used as indicated in the figure.


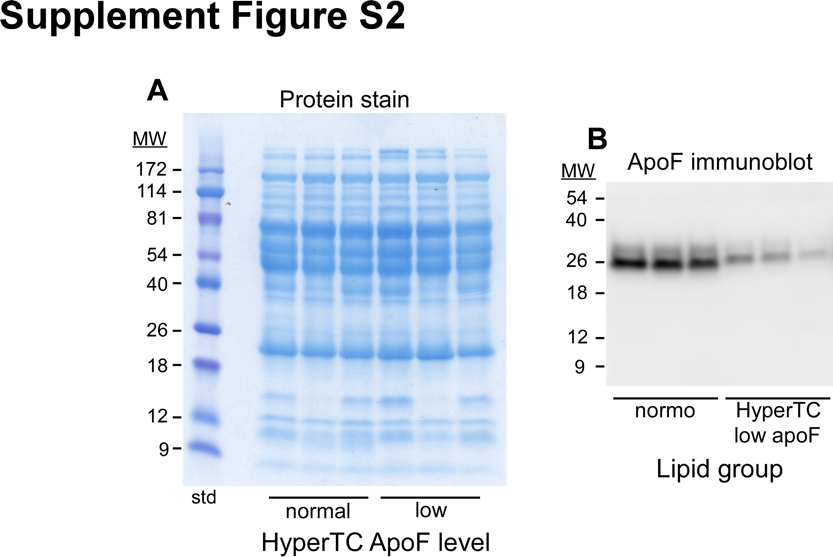


**Supplemental Figure S2** - Analysis of hypercholesterolemic plasmas with very low ApoF content. Panel A: Coomassie blue stained SDS polyacrylamide gel (4-20%) of hypercholesterolemic plasmas with typical levels of ApoF or with very low ApoF levels. Plasmas were depleted of albumin and IgG (LSKMAGD12, MilliporeSigma, Burlington, MA) prior to electrophoresis. Panel B: ApoF western blot of 3 normolipidemic (normo) plasmas and 3 hypercholesterolemic (HyperTC) plasmas with very low ApoF as determined by ELISA.

**References**

1. Day, J. R., J. J. Albers, T. L. Gilbert, T. E. Whitmore, W. J. McConathy, and G. Wolfbauer. 1994. Purification and molecular cloning of human apolipoprotein F. *Biochem. Biophys. Res. Commun.* **203**: 1146-1151.

2. Morton, R. E., H. M. Gnizak, D. J. Greene, K.-H. Cho, and V. M. Paromov. 2008. Lipid transfer inhibitor protein (apolipoprotein F) concentration in normolipidemic and hyperlipidemic subjects. *J. Lipid Res.* **49**: 127-135.

3. He, Y., D. J. Greene, M. Kinter, and R. E. Morton. 2008. Control of cholesteryl ester transfer protein activity by sequestration of lipid transfer inhibitor protein in an inactive complex. *J. Lipid Res.* **49**: 1529-1537.
